# Targeting mailed nicotine patch distribution interventions to rural regions of Canada: protocol for a randomized controlled trial

**DOI:** 10.1186/s12889-020-09810-2

**Published:** 2020-11-23

**Authors:** John A. Cunningham, Michael Chaiton, Scott T. Leatherdale, Alexandra Godinho, Christina Schell

**Affiliations:** 1 Centre for Addiction and Mental Health, 33 Ursula Franklin St, Toronto, Ontario M5S 2S1 Canada; 2 Department of Psychiatry, University of Toronto, Toronto, M5T 1R8 Canada; 3 Research School of Population Health, the Australian National University, Canberra, 2601 Australia; 4 Dalla Lana School of Public Health, University of Toronto, Toronto, M5T 3M7 Canada; 5 School of Public Health and Health Systems, University of Waterloo, Waterloo, N2L 3G1 Canada

**Keywords:** Smoking, Tobacco cessation, Rural, Nicotine patches, NRT, Mass distribution

## Abstract

**Background:**

Quitting smoking is the most effective way of reducing the risk of cancer among smokers. One way of helping people stop smoking is to provide them with free Nicotine Replacement Therapy (NRT), such as when NRT is sent to people by postal mail as part of a mass distribution initiative. Our previous research indicated that there may be a substantial impact on increasing quit rates of a mailed NRT intervention in rural areas. The current research seeks to confirm this finding and to understand the social determinants of health driving these anticipated large effects.

**Methods/design:**

Telephone numbers will be randomly selected from across rural regions of Canada in order to recruit adult smokers interested in completing a smoking survey and willing to be interviewed again in 6 months. The survey will ask participants about their smoking history, demographic characteristics, and a hypothetical question: would they be interested in receiving nicotine patches if they were provided to them free of charge? Half of the smokers interested in receiving nicotine patches will be selected by chance and offered the NRT package. The other half of smokers will not be offered the nicotine patches. In addition, the municipality where each participant lives will be identified and, once the relevant general population data becomes available, attempts will be made to link participant data to relevant municipal characteristics (e.g., smoking rates, availability of health services). Characteristics of the participants and the municipalities in which they live will be used to explain why the nicotine patch intervention may have a larger impact in some rural regions compared to others.

**Discussion:**

The findings from the proposed RCT are timely and of high relevance as the distribution of nicotine patches has substantial potential to combat the public health problem of cigarette related cancer, other diseases, and premature death from tobacco use. Targeting such tobacco cessation initiatives to rural regions may substantially increase the impact of this intervention, helping to optimize the use of limited prevention resources while aiming to save the maximum number of lives.

**Clinical trials registration:**

ClinicalTrials.govNCT04606797, October, 27, 2020.

## Background

In Canada, smoking tobacco accounts for approximately 30% of all cancer deaths and is a risk factor for at least 18 types of cancer [1, 2]. Smoking is also a significant negative contributor to numerous other health conditions [3]. Rates of smoking appear to have recently increased in Canada (15% in 2017 from 13% in 2015) [4]. Cessation plays an impactful role in the prevention of cancer, where 10 years after quitting the mortality rate from lung cancer is about half of that of a continuing smoker [5].

*Rates of smoking are higher in rural than in urban regions of Canada:* Over 6.3 million people live in rural regions of Canada. Tobacco cessation initiatives have been less successful outside of urban areas and provision of health care is challenging in areas of lower population density [6, 7]. Given that Canadian mortality rates are higher in rural than in urban areas [8], and that a significant component of this difference in mortality rates is due to modifiable risk factors, such as smoking, interventions targeting smoking in these regions could reduce these mortality inequalities [9–11]. To reduce the health disparities associated with living in rural areas, new ways of promoting tobacco cessation are needed that are amenable to delivery over large geographic areas. The provision of free nicotine patches sent by postal mail is one such promising option.

*Effectiveness of NRT:* There has been extensive research evaluating the efficacy of NRT as a means to promote smoking cessation. A Cochrane review of 150 randomized trials involving NRT concluded that NRTs increase the rate of quitting smoking by 50 to 70%, irrespective of the clinical setting in which the smoker is treated [12, 13].

*Evidence for the benefits of providing free NRT by postal mail:* There are mass distribution initiatives of free NRT ongoing in several countries, including the USA [14–16] and Canada (Ontario) [17, 18]. Further, there is evidence that the distribution of free NRT is cost-effective, with the cost of $179 per participant in the Canadian mass distribution initiative [18]. Moreover, a promising aspect about mass distribution of NRT initiatives is that, while it is available to everyone, vulnerable populations (whether due to mobility issues, or co-existing mental health concerns) are by far the most likely to respond to the initiative and request free NRT [18]. An additional point as an argument of the benefit of providing free NRT is that it removes a financial barrier to access of NRT for people with low socioeconomic status [19], a factor that often co-occurs with areas of high smoking rates [20]. Thus, the proposed tobacco cessation initiative is an excellent means of targeting vulnerable populations.

*Targeting NRT distribution to regions that may benefit most:* Results from our mailed nicotine patch trial indicated that the impact of free NRT distribution may be unexpectedly large in rural areas (OR = 9.59) [21]. This effect size is substantial in comparison to that observed among participants from urban/suburban regions (OR = 2.65) [22]. While encouraging, this large effect size requires replication to increase confidence in the results because the sample size of participants from rural regions in our earlier trial was small and this was a secondary analysis.

*What other factors might predict a large impact of the NRT intervention?:* Understanding the regional factors driving the impact of the NRT intervention may be essential as it points to a means of targeting limited public health resources to increase the number of people quitting smoking. There is a substantial research tradition investigating individual factors predictive of smoking cessation, including some within the context of RCTs investigating tobacco cessation [23, 24]. Our analytic plan will allow for the consideration of these individual factors alongside other predictors of tobacco cessation. There is less existing research investigating municipal-level factors related to tobacco cessation [20]. It is well known that the prevalence of smoking in adults varies across different provinces and territories of Canada (ranging from 17% in British Columbia to 63% in Nunavut) [10, 25]. Importantly, there are also substantial variations in smoking rates across municipal districts within these larger regions of Canada (e.g., in Ontario, rates of smoking in adults ranged from 15.4 to 44.7% between municipalities, with the highest rates generally in rural municipalities) [20, 26–28]. As a secondary goal of this research, the current project will attempt to nest an RCT within a survey with linked municipal-level data. This multidisciplinary approach should allow us to determine the impact of municipal characteristics on the effectiveness of the NRT intervention.

### Research questions

*Primary question:* Will an intervention of known efficacy – the distribution of free-of-cost nicotine patches sent by postal mail – have a large impact in rural regions of Canada?

*Primary hypothesis:* Participants receiving the NRT package will display significantly greater quit rates (30 day abstinence) at 6-month follow-up as compared to those not offered the NRT package.

*Secondary questions:* What other factors that systematically vary between municipalities impact the effectiveness of the nicotine patch intervention? We will use the work of Corsi et al. [20, 26], as well as work by co-authors Leatherdale and Chaiton identifying geospatial factors related to smoking [29–31], on the community-level factors related to smoking in Canada to identify relevant factors [prevalence of smoking, availability of health services, average socioeconomic status (SES), population density, region of Canada, tobacco retailer density, NRT distributor density (e.g., pharmacy, grocery store)]. We will then attempt to link this general population data to the data collected as part of the RCT. Please note that this linkage will likely occur several years after the completion of the trial because of the usual delay seen between when general population surveys are conducted and when the data is made available to the academic community.

While an extended set of municipal factors listed here will be collected (and examined for their predictive value as part of our secondary analyses), we will focus on two contextual factors that we believe are key to why the NRT intervention may be especially impactful in rural settings.

*Prevalence of smoking hypothesis:* The impact of the NRT intervention will be larger in municipalities where there is a higher prevalence of smoking compared to municipalities where the prevalence of smoking is lower.

*Smoking is more prevalent in rural regions than in urban regions:* [10, 21, 25]. Because of this known higher prevalence of smoking rates in rural versus urban regions, and because the impact of the NRT intervention appears to be unexpectedly large in rural regions, the proposed research predicts that the impact of the mailed nicotine patch intervention will be larger in regions with higher smoking rates versus those in regions with lower smoking rates. There is relevant existing research that supports this postulated differential impact. As smoking rates in an area decline, it has been suggested that those remaining in the population are more hardcore smokers (i.e., less likely to want to quit), whereas in areas with a higher smoking prevalence you are more likely to have smokers in the population who want to quit [32–34].

*Lack of health services hypothesis:* The impact of the NRT intervention will be larger in municipalities where there is less availability of health services compared to municipalities where there is a higher availability of health services.

*Health services* are more difficult to provide in areas of low population density (i.e., rural regions) [6, 7]. The provision of some type of help (in this case a mailed tobacco cessation aid that is well suited for a rural region) will display a larger impact in a situation where there is a lack of other services available than in one that is relatively rich in available health services [35].

## Methods

### Participants

The baseline telephone survey will identify adults (age 18 or over) who smoke 10 or more cigarettes a day, and who are willing to take part in a smoking study that involves two interviews. As part of the baseline survey, all participants will be asked a series of questions to assess their level of interest in receiving free NRT: “The Ministry of Health is considering different ways to help people stop smoking. One option would be to provide interested smokers with free Nicotine Patches. If Nicotine Patches were offered for free, would you be interested in receiving them?” Those who say “yes” will then be asked if they would use the Nicotine Patch to quit smoking. Those who say “yes” to this question will be asked if they would begin to use the Patch within 1 week of receiving it. A “yes” to this question will lead to being asked if they would be willing to have the Patch sent to their home. These items have been employed in our previous research trial of mailed NRT (albeit largely in urban regions) [22]. *Exclusion criteria for randomized trial:* Having a health condition contraindicating NRT use without the supervision of a doctor (i.e. allergy to tape, pregnant or intending to become pregnant, currently breastfeeding, serious heart or circulation problems not including high blood pressure).

### Study design and procedures

**Fig. 1:**
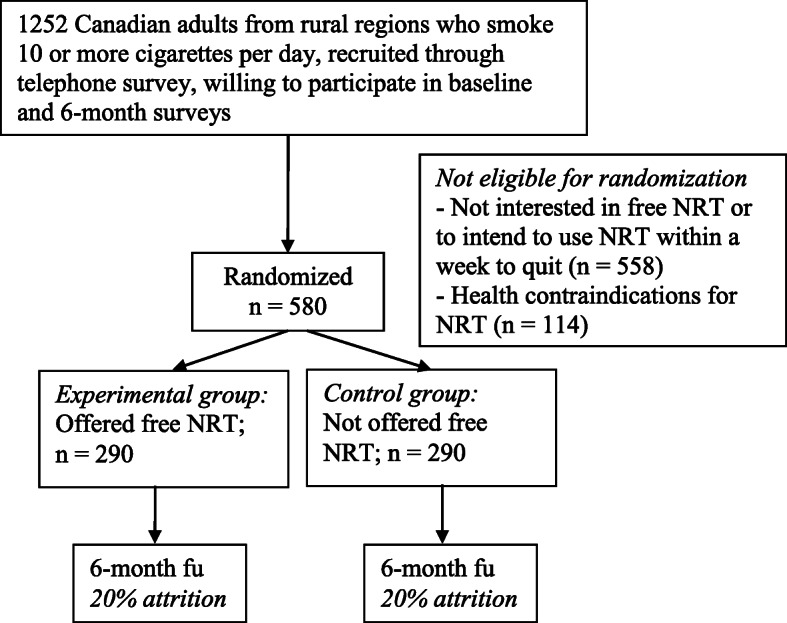
Overview of the proposed intervention trial

The proposed study is a two-arm, single blinded, parallel group randomized controlled trial comparing a mailed nicotine patch intervention to a no intervention control over a 6 month period. A two-stage recruitment process will be employed, in the context of a general population survey with a 6-month follow-up. Random digit dialling of telephone numbers from rural regions of Canada will identify households with adult (age 18 or over) smokers who smoke 10 or more cigarettes a day and who are willing to take part in a smoking study that involves two interviews. Households from rural regions will be defined using the Statistics Canada definition of Rural and Small Town (RST) which are areas with a population of below 1000 and who live outside the main commuting zone of larger urban areas. Participants will be paid $20 for the completion of each of the baseline and 6-month follow-up (payment will be made by cheque – allowing us to collect participant postal codes in order to link participant data to municipal region). Verbal consent will be obtained as the initial contact is by telephone (a copy of the verbal consent script will also be mailed to each participant to keep as a record). Only one person per household will be recruited to participate, using the method where the current smoker with the next birthday in the household is asked to participate. Both cell phone and landline numbers will be contacted. Interviews, and the remainder of the study, will be offered in both English and French. Residence of participants in rural regions will be confirmed by postal code matching. As part of the baseline survey, eligible subjects will be identified for the second recruitment – randomization of smokers into experimental and control conditions to be offered versus not offered Nicotine Patches. A randomized half of the eligible participants will be assigned to the experimental condition and asked for their permission to have Nicotine Patches sent to their home. Participants in the control condition will not be offered Nicotine Patches. See Fig. 1 below for a Consort Diagram summarizing the study design.

### Randomization

Interviews will be conducted using computer assisted telephone interview (CATI) technology. At the end of the baseline interview, eligible subjects will be allocated to experimental and control conditions using block randomization (1:1 ratio) built into the CATI program (no stratification or minimization is necessary given the large proposed sample size in this trial).

*Protecting against sources of bias:* The interviewers conducting the baseline and 6-month post-intervention follow-up survey will be blind to experimental condition at the time the key outcome questions are asked. Further, while it is possible that a participant might ‘unblind’ themselves to an interviewer by volunteering that they used the nicotine patch, this is still unlikely to have an impact as the interviewers will be part of a separately contracted telephone survey research firm and will not be aware of the hypotheses of this research trial or be involved in the randomization process.

### Ethical approval

This study, including methods and design, has been approved by the standing ethics review committee of the Centre for Addiction and Mental Health (CAMH – REB Protocol #006/2020).

### Interventions

*Intervention group:* Participants randomized to the intervention group will be asked, at the end of the baseline survey, if they want to be sent a free, 5-week supply of Nicotine Patches (“As part of a pilot trial, the Centre for Addiction and Mental Health has a supply of Nicotine Patches to distribute to interested smokers. You told us that you would be interested in receiving a free supply of Nicotine Patches. Do we have your permission to mail them directly to you at your home?”). Those agreeing will have the NRT mailed to their home the week after their baseline interview. A 5-week program of Nicotine Patches will be sent (3 weeks of Step 1 [21 mg of nicotine]; 1 week of Step 2 [14 mg of nicotine]; 1 week of Step 3 [7 mg of nicotine]). The mail-out will also contain a letter that advises participants to talk to their doctor if they have questions or concerns about the use of NRT. Once randomly assigned to this condition, participants will be counted as part of the intervention group whether they agree to accept the NRT or not (intent-to-treat approach).

*Control group:* Participants randomly assigned to the control group will not be offered Nicotine Patches at the end of the baseline survey. An advantage of the design procedure to be employed in this trial is that participants in the control group will not have the expectation that they will receive NRT. Thus, their smoking outcomes at 6-month post-intervention follow-up will reflect a true natural history comparison to the outcomes of participants in the experimental group.

### Measures

The schedule of enrolment, intervention and assessment are presented in Table 1.

**Table 1:** Schedule of enrolment, intervention and assessment

Study periods	Inclusion procedure	Baseline assessment	Follow-up
Time	0	0	6
Expected duration (minutes)	3	12	10
Inclusion criteria	x		
Patient Information and Informed Consent	x		
Demographics (questionnaire)	x	x	
Primary outcomes variables (questionnaires)		x	x
Secondary outcomes variables (questionnaires)		x	x
Other outcomes of interest (questionnaires)		x	x
Randomization		x	
Intervention group		NRT package mailed after baseline assessment	

*Primary outcome measures:* The primary outcome measure will be 30-day point prevalence abstinence at 6 months post-intervention. This was also the primary outcome measure in our original trial and is a recommended outcome variable for tobacco cessation trials [22]. Continued use of the same outcome variable allows us to merge the two datasets to allow an urban comparison group for the proposed trial.

*Content of baseline survey:* All items have been employed by us in previous studies, including our earlier RCT of mailed NRT in a largely urban sample [22]. Participants will be asked: a) number of cigarettes smoked per day; b) level of nicotine dependence using the revised Fagerstrom test for nicotine dependence (Test-retest reliability = .78–.84) [36, 37]; c) number and duration of past quit attempts; d) past use of NRT and other anti-smoking medications; e) use of stop smoking services; f) intent to quit smoking in the next 6 months and 30 days; g) quantity and frequency of alcohol consumption; h) illicit drug consumption (ever and past year); and i) a series of demographic characteristics. Items assessing the inclusion criterion of interest in receiving nicotine patches will be nested within items assessing use of stop smoking medications. Finally, questions about general health status will be asked which will include questions about health contraindications for nicotine patch use.

*Content of 6-month post-intervention follow-up survey:* Participants will be asked their current smoking status, including length of time without smoking in order to assess the primary outcome variable. Participants who have not quit will be asked how many cigarettes per day they currently smoke and their intentions to quit smoking; how soon after waking they smoke their first cigarette; and if, since their baseline interview, they have stopped smoking for even 1 day because they were trying to quit. Participants in the experimental group will be asked if they received free NRT and, if they did, to evaluate their experiences with NRT and to elaborate on their efforts to quit smoking. These questions will include items asking whether the participants used the NRT (none, some, all) and what they did with the NRT that they did not use themselves. These questions will be asked after the core smoking status outcome measures have been assessed. Participants in the control group will also be asked about their use of NRT but the questions will be framed to reflect purchase of NRT from sources other than this ongoing study. Participants in both conditions will also be asked about their use of other smoking cessation aids and services, including electronic cigarettes, self-help/online methods, as well as participation and/or receipt of various forms of advice and counselling from family physicians, smoking cessation specialists, or other healthcare providers. As some individuals are known to initiate use of other tobacco products after stopping use of conventional cigarettes, such as cigars or non-combustible products such as smokeless tobacco or electronic cigarettes, participants who report smoking abstinence will be asked if they currently use other tobacco or nicotine containing products. This will allow us to examine whether people who quit cigarettes compensate with other forms of nicotine delivery.

*Linking to municipal characteristics data:* Attempts will be made to access relevant municipal characteristics for the proposed analyses from the most recently available Canadian Community Health Survey (CCHS) data sets or from other general population data sets with relevant variables (average smoking rate, region, population density, average SES). If successful, municipal characteristics will be merged with the baseline and follow-up data collected as part of this trial. We will also attempt to access health services availability using publicly available data on number of MDs by location, as well as other public health services locations. The approach will be similar to that outlined by McEachern et al. [38]. For the proposed trial, we plan to employ the database of Desktop Mapping Technologies Inc. (DMTI) [39]. Enhanced Points of Interest (EPOI) is a vector GIS database of over 1 million business and recreational points of interest for all provinces/ territories in Canada (e.g., health care facilities, tobacco retailers, pharmacies). Data linkage to the participating municipalities will be consistent with linkage approaches used in previous research where geocodes for the relevant businesses are located in each municipal buffer (i.e., bounded areas for each municipality in which the different built environment characteristics are quantified) [40, 41]. Distance between participants’ location and the closest health services available will be recorded.

### Power calculations

The power calculation was conducted to estimate the sample size needed to test the primary hypothesis that participants receiving the NRT package will display significantly greater quit rates (30 day abstinence) at 6-month follow-up as compared to those not offered the NRT package. Our secondary analysis of the rural participants in our earlier trial indicated a large effect (OR = 9.59; 8.9% 30-day abstinence rate at 6 months in those receiving NRT vs 1% in those not receiving the intervention). However, this estimate is not stable due to the small sample size (*n* = 200 in participants from rural regions; 95% CI 1.19 to 77.16). As such, we have reduced the anticipated size of the impact of the intervention to 75% to generate the sample size estimate for the current proposal (i.e., OR = 7.1; 6.7% 30-day abstinence rate at 6 months in those receiving NRT vs 1% in those not receiving the intervention). This will allow for the likely possibility that, while the impact of the NRT intervention is large in rural regions, it may not be as large as was observed in the secondary analyses. The power analysis was based on Monte Carlo simulations. Randomly distributed dropouts were also included in the simulations, at a rate of 20%; thus accounting for the 20% attrition expected between baseline and 6-month post-intervention follow-up based on earlier trial findings [22]. Quitting smoking was the binary outcome, measured as 30-days abstinence. A sample of 290 participants per group (intervention versus control) is needed in order to obtain 80% power with 95% confidence interval. Further, analyses from our earlier trial established that 50.1% of participants from rural regions would be interested in receiving nicotine patches (and would use it within a week to quit), leading to a baseline survey needed of 1138 participants. Finally, results from our earlier trial found that approximately 10% of those recruited at baseline were not eligible because of reporting a health condition contraindicating NRT use without the supervision of a doctor. Thus, in order to conduct this trial, we would need to recruit 1252 participants for the baseline survey in order to have sufficient participants for the trial (1138 X 110%).

### Analysis plan

Multilevel logistic modelling will be employed. The analyses are staged, with tests of the primary hypothesis (impact of the NRT intervention on 30-day abstinence rates at 6 months) as the first step and with step 2 involving tests of municipal- and individual-level factors predicted to explain the anticipated large impact of the NRT intervention in rural settings. Please note that the step 2 analyses will be published separately from the step 1, primary analyses. This is because the general population data necessary to generate the municipal-level data will likely not be available until several years after the completion of this trial.

#### Step 1

Tests whether the provision of the NRT intervention impacts on 30-day abstinence rates. Experimental condition (participant randomly assigned to receive the NRT intervention package or to the control condition) is the categorical predictor in these analyses. Participants lost to follow-up will be assumed to be current smokers. As part of the step 1 analyses, we will also conduct a chi-square test to explore whether there is differential loss to follow-up between experimental conditions. Further we will replicate these analyses with all subjects with missing data excluded from the analyses. If the results are the same between the analyses where the missing data is excluded and where subjects lost to follow-up are assumed to still be current smokers, then we can safely assume that there is no differential attrition between conditions (or, at least none that impacted on the outcome of the trial).

*Step 1 secondary analyses:* Additional secondary analyses will assess the effects of NRT and other predictors on relevant dependent measures pertaining to those who have not quit. These analyses will include multilevel models predicting the number of cigarettes smoked, intentions to quit, and whether participants have quit temporarily during a given time-period.

#### Step 2

Tests to identify factors that systematically vary between individuals, and between municipalities, that explain the size of the impact of the NRT intervention on self-reported 30-day abstinence at 6-month follow-up. For these analyses, the data from the urban comparison sample [22] will be combined with the data collected in the current project of rural participants in order to allow for stronger tests. We have *pre-specified two hypothesized municipal factors* and predict that prevalence of smoking at the municipal level, and availability of health services in the municipality, will be related to the size of the impact of the NRT intervention. The multi-level logistic regression models constructed will include municipal-level sampling frame data (province or territory region of Canada), and municipal level smoking prevalence and health services availability data to determine the size of the impact of the intervention with the individual-level outcome data. Individual demographic (including participant gender) and severity of smoking characteristics will be included in order to identify individual-level factors relevant to the impact of the intervention.

*Stage 2 secondary analyses* will add other characteristics of the municipality [average SES, population density, tobacco retailer density, NRT distributor (e.g., pharmacy, grocery store) density] to identify other contextual factors that may be important in determining the size of the impact of the NRT intervention.

## Discussion

Preventing cancer through reducing smoking rates is a cornerstone of improving the health of Canadians. Mass distributions of nicotine patches have become a major prevention initiative in Canada. The proposed trial will combine an RCT with a municipal linking strategy in order to systematically study how social and environmental factors that vary at the municipal-level will impact on the efficacy of this health intervention. What if the significant resources devoted to nicotine patch distribution where better targeted to regions where they could have the greatest impact and further, help address the health disparities associated with rural regions of Canada? This trial will allow us to answer this question, promising greater success in preventing cancer through promoting tobacco cessation.

### Trial status

Protocol version: 1.

Date recruitment will begin: November 2020.

Approximate date recruitment will be completed: August 2021.

## Data Availability

Not applicable.

## Abbreviations

RCT: Randomized controlled trial
NRT: Nicotine replacement therapy
